# Influence of Forced Online Distance Education During the COVID-19 Pandemic on the Perceived Stress of Postsecondary Students: Cross-sectional Study

**DOI:** 10.2196/30778

**Published:** 2022-03-15

**Authors:** Andrej Šorgo, Nuša Crnkovič, Branko Gabrovec, Katarina Cesar, Špela Selak

**Affiliations:** 1 Faculty of Natural Science and Mathematics University of Maribor Maribor Slovenia; 2 National Institute of Public Health Ljubljana Slovenia

**Keywords:** online study, stress, COVID-19, postsecondary students, pandemic, epidemiology, educational institutions, online education, pedagogy, mental health

## Abstract

**Background:**

One of the most significant changes in the majority of postsecondary educational institutions was the closure of those institutions and the shift of educational activities to online distance learning formats as a result of the COVID-19 pandemic. Closure combined with forced online distance education (FODE) was a cure with many side effects, 1 of them being the effect on students’ mental health and, more specifically, levels of stress. Due to the novelty of the situation, there have been no studies so far designed to link satisfaction with online study, feelings toward the study obligations, and stress among students.

**Objective:**

The aim of the study is to assess the perceived stress of Slovenian postsecondary students in order to identify the online study–related factors affecting or acting as a covariate during the COVID-19 lockdown.

**Methods:**

Data collection was conducted through a self-reported survey as part of a large cross-sectional study based on data collected from postsecondary students from a number of higher educational institutions. The random sample consisted of 4455 individuals. The Perceived Stress Scale (PSS-4), Satisfaction with Online Study Scale (SAT-5), and Feelings Towards Study Obligations Scale (FETSOS) were used to assess the constructs and the relations observed within the study.

**Results:**

The results indicate that more than half of all respondents reported high levels of stress. The difference in the reported levels of perceived stress between genders were statistically significant (N=4454, *F_2_*=56.719, *P*<.001, Cohen *d*=0.35). Overall, the results suggest that a decline in the motivation to study, the quality of internet and mobile connections, and the presence of distracting factors in the study space were the 3 main factors related to the students’ negative emotions as associated with the timeliness, performance, and quality of the study obligations. Furthermore, the results show that the level of satisfaction with online study affected stress such that the higher the satisfaction, the lower the stress. Moreover, the more positive feelings connected with the timeliness, performance, and quality of the study obligations that the students felt, the more satisfaction they reported with online study and, thus indirectly, lower stress and less negative feelings.

**Conclusions:**

The findings of this study call for implementing structures and measures targeted at stress reduction, working conditions, and pedagogy with regard to FODE.

## Introduction

Academic environments have always been known for the presence of stress, anxiety, and depression [1,2]. The closure of postsecondary educational institutions induced by COVID-19 brought about many changes to almost all areas of study. In this context, study mostly takes place online and at a distance. Because of its compulsory nature, it has been recognized as forced distance education (teaching, learning) and in its online form as forced online distance education (FODE) [3]. Moving lectures and courses online has not only changed the format of courses but also brought with it several side effects [4]. One of the most often reported side effects of FODE is the impact on students’ mental health, with stress, anxiety, and depression being the most commonly reported impacts worldwide [5-7], with some subpopulations being more vulnerable than others [8]. Students seem to be at greater risk of mental health problems due to social distancing, as well as FODE and other measures to prevent or reduce the risk of transmission of coronavirus (SARS-CoV-2), such as restrictions on movement and gatherings [9]. Previous research has shown a link between psychological distress and symptoms of mental disorders or problems with outbreaks of infectious diseases [10,11]. Research has shown that during the COVID-19 pandemic also, psychological reactions, such as stress, anxiety, and depression, have been common [12,13] among students as well [14-21]. Several studies have reported high or increased prevalence of stress among this population [6,8,17,19,22,23], associated with factors such as fear and worry about one’s own health or that of loved ones, difficulties concentrating, disrupted sleeping patterns, reduced social interaction, and increased concerns about academic performance [19], which raises concerns about the mental health of postsecondary students.

The studies reviewed most often searched for predictors of increased mental health problems during a lockdown in socioeconomic domains, previous (mental) health episodes, living conditions, and, more rarely, the domain of education [24-26]. The majority of studies, produced by professionals from the fields of psychology and psychiatry, most often suggest solutions for building resilience among students, counselling, and, at the last instance, medication [8,27]. However, the solution for students can lie not only in improving self-supportive and institutionally supported measures for those with mental health problems but also in reducing sources of stress where and when possible. However, many studies of online distant (remote) education in the educational research domain are from the pre-COVID-19 era. The pandemic has put these previous findings into a new context. Those pre-COVID-19 findings cannot be easily transferred, because the earlier studies were based mostly on voluntary decisions, while at least during the first lockdown, the transition was forced and not tailored to the best standards of remote education. In the words of Hodges et al [28], “Everyone involved in this abrupt migration to online learning must realize that these crises and disasters also create disruptions to student, staff, and faculty lives, outside their association with the university.”

In recent research, perceived stress has been measured as an output variable, as a covariate with several manifestations of mental health problems, such as anxiety and depression (among numerous others), commonly associated with pandemic measures [12,29]. The input constructs included factors extracted from the exploratory analysis of variables believed to be stressors produced in relation to learning experiences, and thus can be manipulated by educational institutions and educators. As an intellectual framework, we followed the Sternberg theory of successful intelligence [30], as elaborated for FODE in Dolenc et al [4], which claims that educators “have the choice of adapting to the new environment, adapting the environment or changing the environment, while students can only adapt to the environment.”

Postsecondary students can be regarded as 1 of the most important investments of every society in its future prosperity [1]. It is obvious that the share of individuals with higher education should and will rise in the coming generations [31]. There is also a trend toward the digital transformation of education, with the transition to more or less blended forms of education with promises of “anywhere (any place) – any time” learning experiences [32,33]. These trends call not only for an increase in the number of study places and the digital transformation of communication channels at educational institutions (colleges, higher schools, academies, faculties, universities, etc) but also for greater support for student well-being. In the transitional period between adolescence and adulthood, postsecondary educational institutions should be concerned with the quality of the learning outcomes of diverse student populations in line with student health and well-being. As such, the findings and experiences from the COVID-19 lockdowns should not be forgotten but should be taken not only as an opportunity to identify problems that may have been masked and unacknowledged before the lockdown but also as an incentive to address them. In line with these intentions, the first part of this study is descriptive, with the second part suggesting prescriptive measures.

The aim of the study was twofold. The primary focus was on assessing the mental health of Slovenian postsecondary students and identifying the online study–related factors affecting or acting as a covariate with it during the COVID-19 lockdown. The data used in this study were collected from postsecondary students from various higher educational institutions as part of a project titled “Measures in the Field of COVID-19 Spread Management With a Focus on Vulnerable Populations” [34]. The assumptions of the study were that technology was not neutral and that satisfaction with online experiences and with forced and involuntary study conditions during the lockdown could work as an incubator to raise perceived stress, as shown in previous studies [34]. The authors are aware that a number of factors and variables may correlate with, be influenced by, or predict stress. Among others, we can list (in no particular order) anxiety, depression, resilience, fear of COVID-19, previous mental illness, substance abuse, and addiction. However, at this point, such relationships were not explored. This was not because they were unimportant but because the primary goal was to explore the effects of FODE on stress within a simple and robust model.

The study was divided into 2 parts, in line with the research questions (RQs):

- RQ1: What are the latent structure, reliability, and construct validity of the instruments used in the survey?
- RQ2: What are the strength and direction of paths between latent variables?

The study results may help us produce prescriptive measures to help improve study conditions and the mental health of postsecondary students and also help those who may be concerned with the problem and have the ability and means to take action during and after the COVID-19 pandemic.

## Methods

### Procedures and Instruments

Data collection was conducted through a self-reported survey as part of a large cross-sectional study in Slovenia. The data collection took place between February 9 and March 8, 2021. Data collection was conducted through the web-based survey platform 1KA (Centre for Social Informatics, at the Faculty of Social Sciences, University of Ljubljana) [35]. Simple random sampling was used, and invitation letters to participate in the study were sent by email to all universities, private faculties, and student organizations, with a request to forward the invitation to all their students. To obtain as much feedback as possible, a reminder letter with the invitation to participate was sent to all addresses after 1 week and, after another week, to those from whom we had not received any feedback. Respondents were informed about the various aspects of the study, including their right to voluntarily participate in and withdraw from it.

An online questionnaire (see Multimedia Appendix 1) was used for data collection. In addition to demographic data, metric tools were used to assess perceived stress, satisfaction with online study, and feelings toward the study obligations.

The Slovenian translation of the Perceived Stress Scale (PSS-4; see Multimedia Appendix 2), a shortened version of PSS-10 and PSS-14, was used for the assessment of psychological stress [36-38]. The items in the instrument ask respondents to report on how they have coped with various situations over the past month. The instrument has 4 items, 2 of which (items STR2 and STR3) have a reverse score. The response format is 0=never; 1=almost never; 2=sometimes; 3=fairly often; and 4=very often, with higher totals indicating higher levels of stress (the scores range from 0 to 16). The perceived stress level was categorized as low versus high perceived stress based on a median split (<8 vs ≥8) in analogy with application of the PSS-10 instrument in the Slovenian sample [39].

The Satisfaction with Online Study Scale (SAT-5; see Multimedia Appendix 3), initially developed by Debevc et al [40] and adapted by Ploj-Virtič et al [3], was used for assessment of satisfaction with online study. The scale is rooted in flow theory [41] and was applied in slightly different versions [3,42]. The measurement encompassed a 7-point Likert scale ranging from 1=strongly disagree to 7=strongly agree, with a total score ranging from 5 to 35 and higher scores indicating higher perceived satisfaction. The scale shows unidimensionality and Cronbach *α* values >.80 in all studies in which it was used.

The severity of several factors inducing negative feelings connected with the timeliness, performance, and quality of the study obligations were assessed using the Feelings Toward Study Obligations Scale (FETSOS; see Multimedia Appendix 4). The scale was designed for the purpose of this study. The authors consulted the outcomes of the study by Dolenc et al [4] and transferred some of their findings into statements (items) of the scale. The response format was 1=no impact; 2=very weak impact; 3=weak impact; 4=moderate impact; 5=strong impact; 6=very strong impact; and 7=absolute impact. The scale has 12 items. Because of their diversity, it was not expected to be unidimensional, which was later confirmed. Theoretically, the span of the scale is between 12 and 84, with higher numbers indicating more negative impacts. Three subscales can be identified: descriptors of working conditions, descriptors of pedagogy, and descriptors of well-being and health.

Quantitative measures of all subscales are provided in the Multimedia Appendices.

### Ethics Approval

Ethical approval to conduct the study was obtained from the National Medical Ethics Committee of the Republic of Slovenia (NMEC), Ministry of Health (no. 0120-48/2021/3).

### Statistical Analyses

We collected responses from 5999 full-time students. Due to the planned analyses, attrition, and random missing responses, the database was cleared and only data sets from those individuals who provided full responses for all the constructs were selected (4455/5999, 74.26%). We calculated the frequencies and measures of central tendencies for each item and the sums of items, when appropriate. Factor analysis was conducted to examine and validate the factor structure of the data matrix of the scales. In the factor analysis, the total sample (N=4455) was randomly divided into an exploratory factor analysis (EFA) sample (n=2235, 50.17%) and a confirmatory factor analysis (CFA) sample (n=2220, 49.83%). EFA was conducted to examine the underlying factor structure of the constructs in the EFA sample using principal axis factoring (PAF) analysis. Because correlations between components were expected, direct oblimin rotations were applied. Parallel analysis was used to determine the number of factors extracted from the EFA [43]. Cronbach *α*>.70 and unidimensionality of the construct were the entrance criteria to be included in CFA.

Two approaches were used for building the models. The first was based on correlations between the sums of the extracted constructs, where a path direction was not guessed. Spearman *ρ* was used for this. The second model was based on structural equation modeling (SEM) analysis [44,45], where hypothesized models were tested to fit the data. The maximum likelihood method was used, and analysis of the residual covariance matrix and inspection of the modification indices were applied to improve model fits. The whole data set (N=4455) was included in the final SEM analyses. We chose a selection of fit indexes, as proposed by Gaskin and Lim [46], and applied it using the IBM AMOS plugin proposed by Hu and Bentler [47]. The cut-off criteria recognized as acceptable are as follows: comparative fit index (CFI)>0.90, standardized root-mean-square residual (SRMR)<0.08, and root-mean-square error of approximation (RMSEA)<0.06. CFA with maximum likelihood estimation [48] was used for model fitting with the application of IBM AMOS 27 and IBM SPSS Statistics 27 for EFA.

### Research Model

The hypothesized research models were based on the following hypothesized paths:

- Hypothesis 1 (H1): Level of satisfaction with online study influences (correlates with) stress.
- H2: Feelings toward online study (working conditions, pedagogy, and well-being) influence (correlate with) satisfaction.
- H3: Feelings toward online study (working conditions, pedagogy, and well-being) influence (correlate with) stress.

**Figure 1:**
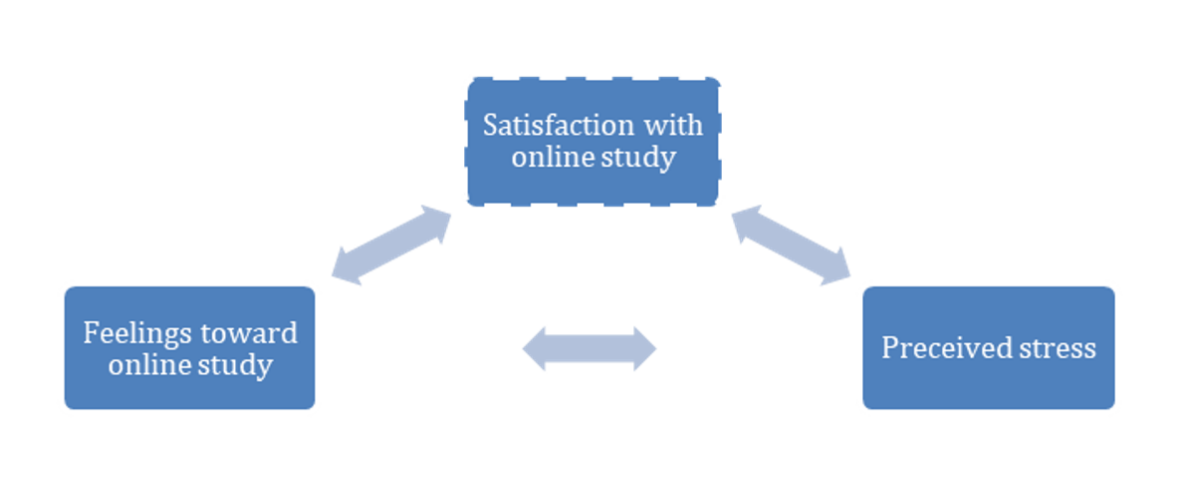
Framework of the study.

It was assumed that satisfaction would work as a mediator between feelings toward online study (working conditions, pedagogy, and well-being) and stress (Figure 1).

## Results

### Characteristics of the Survey Respondents

The demographic characteristics of the students (Table 1) included in the study show there were more males (n=3234, 72.59%) than females included in the sample and more than half of the students were single (n=2467, 55.38%). The majority were bachelor’s degree students (n=2696, 60.52%). The most numerous study fields were health and medicine (n=886, 19.89%), science and mathematics (n=871, 19.55%), noneducational social studies (n=801, 17.98%), and humanities (n=707, 15.87%).

**Table 1:** Sample characteristics (N=4455).

Demographic characteristics		Frequency, n (%)
**Gender**		
	Male	3234 (72.59)
	Female	1186 (26.62)
	Other	34 (0.76)
	Missing	1 (0.02)
**Educational level**		
	Higher vocational	129 (2.90)
	Bachelor’s study	2696 (60.52)
	Master’s study	1606 (36.05)
	Doctoral study	18 (0.40)
	Other	6 (0.13)
**Relationship**		
	Single	2467 (55.38)
	In a relationship	1919 (43.08)
	Other	68 (1.53)
	Missing	1 (0.02)
**Field of study**		
	Health and medicine	886 (19.89)
	Science and mathematics	871 (19.55)
	Social studies (noneducation)	801 (17.98)
	Humanities	707 (15.87)
	Art	409 (9.18)
	Technology and engineering	369 (8.28)
	Education	251 (5.63)
	Security	128 (2.87)
	Other	33 (0.74)

### Latent Structure, Reliability, and Construct Validity of the Instruments

EFA of PSS-4 (n=2235, 50.17%) revealed that it is a unidimensional factor (latent variable), with Cronbach *α*=.80. The explained variance was 50.3% (eigenvalue=2.496). Measures of the central tendencies of 2 positively (reversed) and 2 negatively worded items, as well of the results of PAF, can be seen in Multimedia Appendix 2.

CFA (n=2220, 49.83%) was performed with 1- and 2-factor models. The difference is that in the 1-factor model, all 4 items load on a single factor, whereas in the 2-factor model, the positively loaded items load on the first factor and the negatively loaded items load on the second factor. A 1-factor solution, even if the 2-factor model shows a slightly better fit, was chosen to be included in the models used to predict the hypotheses. The reason was that we wished to include PSS-4 as a complete and valid instrument. The values of the FIT indexes with constrained STR2 and STR3 (r=0.38) were as follows: chi-square (CMIN)=31.824, *df*=1, CMIN/*df*=31.824, CFI=0.995, SRMR=0.013, and RMSEA=0.081.

According to EFA, SAT-5 is a unidimensional tool (Cronbach *α*=.88), and the first factor (eigenvalue=3.399) explained 67.971% of the variance. The values of the FIT indexes for the SAT-5 were as follows: CMIN=36.181, *df*=3, CMIN/*df*=12.060, CFI=0.995, SRMR=0.016, and RMSEA=0.071. Constrained pairs of error terms were between SAT2 and SAT5 (r=–0.36) and SAT3 and SAT4 (r=–0.22). Measures of central tendencies, communalities, and factor loadings of SAT-5 are presented in Multimedia Appendix 3.

Analysis of the results obtained by FETSOS (Cronbach *α*=.88) revealed a 2-factor structure, cumulatively explaining 56.30% of the variance. The first factor included all 12 listed items (eigenvalue=5.295, variance=44.126%, Cronbach α=.88), while the second factor consisted of 2 items (equipment, and mobile and internet connections) negatively cross-loading to the first factor as well (eigenvalue=1.461, variance=12.173, Cronbach *α*=.90; see Multimedia Appendix 4). The structure obtained did not follow the theoretically predicted subscales (pedagogy, working conditions, and well-being; see Multimedia Appendix 4). Measures of central tendencies, communalities, and factor loadings of FETSOS are presented in Multimedia Appendix 4.

### Differences of Perceived Stress, Satisfaction With Online Study, and Feelings Toward Study Obligations Between Genders and Study Enrolment Levels

Analysis of perceived stress level categorized as low versus high perceived stress based on a median split (<8 vs ≥8; N=4455) revealed that the sample mean was 7.97 (SD 3.32) and that 1938 (43.50%) of the 4455 students belonged to the lower-stress group and more than half of them to the high-stress group. The upper quarter (≥13) contained 412 (9.25%) of the 4455 students.

The difference in the means of the reported levels of perceived stress (Table 2) between genders were statistically significant, with the highest for those who reported a nonbinary gender, followed by females, both above the median split. Males were below the median split. The effect size between males and females could be regarded as a small effect.

When comparing students from the 2 educational levels (Table 3), it appeared that those studying for a bachelor’s degree experienced a higher level of stress than those studying for a master’s degree or a doctorate. The effect size between both levels, calculated as Cohen *d*, could be regarded as a small effect.

Regarding satisfaction with online study, an examination of the measures of central tendencies (see Multimedia Appendix 3) revealed that most of the students thought that their online experiences were comprehensible, successful, and instructive but not easy or entertaining.

The difference between genders based on the means of the responses to SAT-5 was not statistically significant (N=4454, *F*_2_=0.18, *P*=.98). Therefore, the results are not reported in the table.

**Table 2:** Differences between genders for PSS-4^a^ and FETSOS^b^ scores (N=4454).

Gender	PSS-4 (*F*_2_=56.719, *P*≤.001, Cohen *d*=0.35 [95% CI=0.284-0.418])		FETSOS (*F*_2_=74.771, *P*≤.001, Cohen *d*=–0.412 [95% CI=0.345-0.479])	
	Mean (SD)	95% CI	Mean (SD)	95% CI
Men	7.12 (3.31)	6.93-7.30	49.18 (15.32)	48.30-50.05
Women	8.27 (3.26)	8.16-8.39	55.13 (14.109)	54.65-55.62
Nonbinary	9.21 (3.18)	8.10-10.32	57.03 (15.15)	51.74-62.32

^a^PSS-4: Perceived Stress Scale.

^b^FETSOS: Feelings Towards Study Obligations Scale.

**Table 3:** Differences between educational levels for PSS-4^a^, SAT-5^b^, and FETSOS^c^ scores (N=4467).

Educational level	PSS-4 (*F*_1_=27.201, *P*≤.001, Cohen *d*=0.164 [95% CI=–0.103 to 0.225])			SAT-5 (*F*_1_=4.231, *P*=.04, Cohen *d*=0.064 [95% CI=0.003-0.125])			FETSOS (*F*_1_=9.889, *P*=.002, Cohen *d*=0.097 [95% CI=0.037-0.158])		
	n (%)	Mean (SD)	95% CI	n (%)	Mean (SD)	95% CI	n (%)	Mean (SD)	95% CI
Bachelor’s study	2825 (63.24)	8.17 (3.31)	8.05-8.29	2825 (63.24)	17.77 (8.02)	17.48-18.07	2825 (63.24)	54.08 (14.791)	53.54-54.63
Master’s or doctoral study	1642 (36.76)	7.63 (3.26)	7.47-7.79	1642 (36.76)	18.27 (7.43)	17.91-18.46	1642 (36.76)	52.65 (14.458)	51.94-53.35

^a^PSS-4: Perceived Stress Scale.

^b^SAT-5: Satisfaction with Online Study Scale.

^c^FETSOS: Feelings Towards Study Obligations Scale.

When comparing students from the 2 educational levels, it appeared that those from the bachelor’s level were less satisfied than students from the master’s or doctoral levels (Table 3). The effect size between both levels was negligible, which can, in practice, be regarded as the absence of an effect. Furthermore, regarding feelings toward the study obligations, according to the opinions of the students (see Multimedia Appendix 4), the top 3 items that negatively influenced their feelings connected with the timeliness, performance, and quality of the study obligations are (1) a decline in the motivation to study, (2) quality of internet and mobile connections, and (3) the presence of distractions in the study space (eg, other people). In contrast, the 3 items with the least negative impact were (1) the need to earn an income, (2) health problems directly related to distance learning, and (3) health problems not directly related to distance learning.

The difference between genders based on the means of the responses were statistically significant, with the highest for those who reported a nonbinary gender, followed by females and males (Table 2). From the SD values, a huge variation in the responses could be observed. The effect size between males and females, calculated as Cohen *d*, could be regarded as a small effect.

When comparing students from 2 educational levels, it appeared that those from the bachelor’s level reported overall higher negative feelings than students from the master’s or doctoral levels (Table 3). The effect size between both levels, calculated as Cohen *d*, could be regarded as a small effect.

### Strength and Direction of Paths Between Latent Variables

Correlation analysis between sums of scales (Table 4) showed that negative feelings toward online study (FETSOS) moderately and negatively correlated with (SAT-5) and moderately correlated with stress (PSS-4).

**Figure 2:**
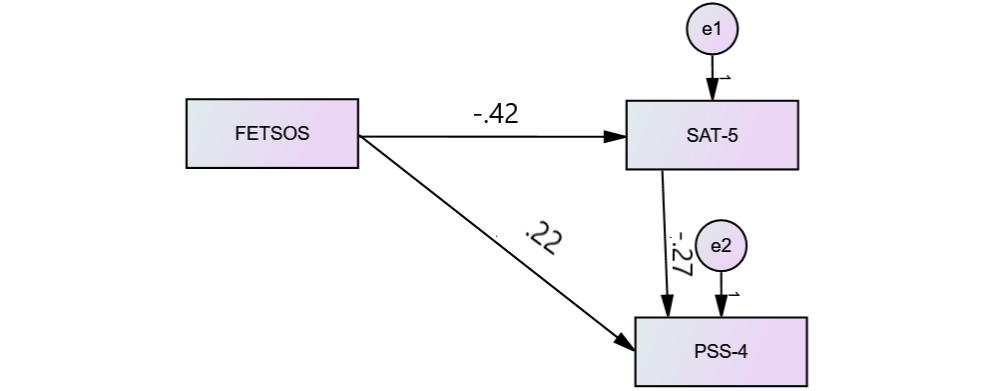
Path coefficients between sums of scales. FETSOS: Feelings Towards Study Obligations Scale; PSS-4: Perceived Stress Scale; SAT-5: Satisfaction with Online Study Scale.

Furthermore, the results of SEM analysis with SAT as a moderator variable (Figure 2) revealed that 18% of the variance of SAT-5 could be explained by FETSOS and 17% of the variance of stress as the main outcome variable could be explained by the joint influence of FETSOS and SAT-5. The values explaining model fits were CFI=1, SRMR=0, and RMSEA=0.359, which led us to build alternative models.

**Figure 3:**
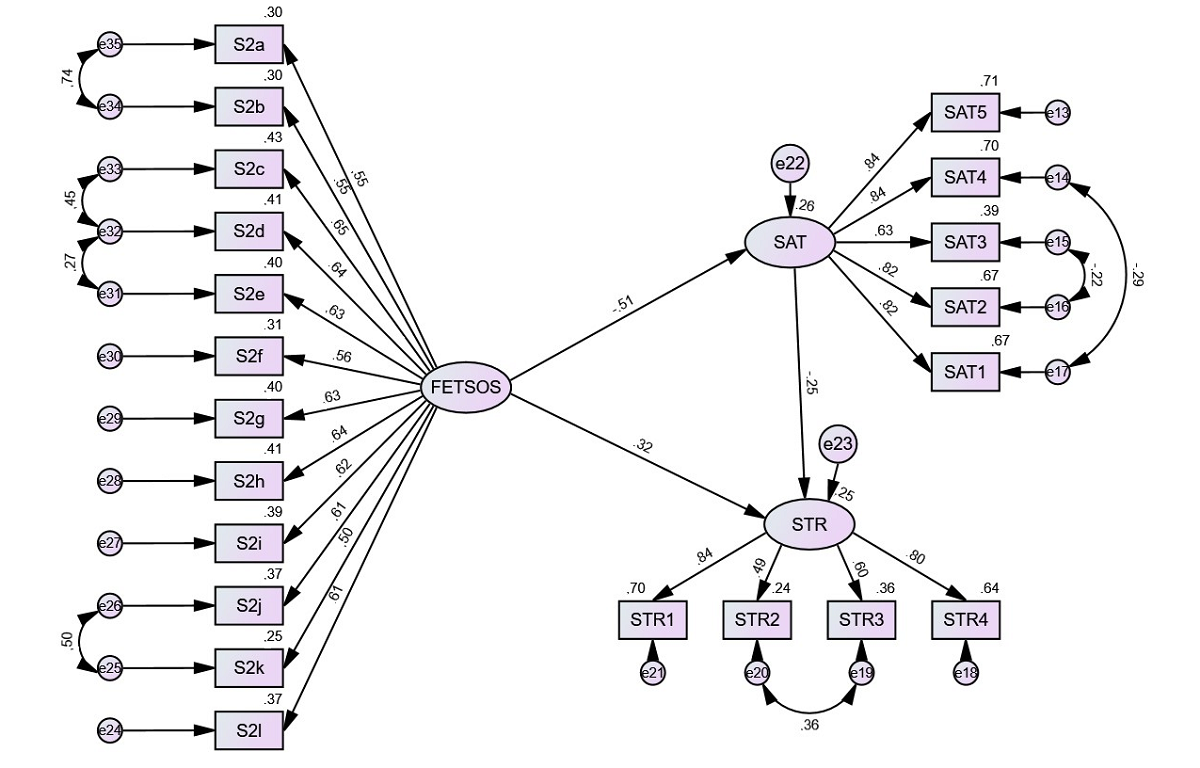
Measurement and structural model connecting FETSOS (variables S2a-S2l), SAT-5, and STR (PSS-4). FETSOS: Feelings Towards Study Obligations Scale; PSS-4: Perceived Stress Scale; SAT-5: Satisfaction with Online Study Scale.

The hypothetical model followed all 3 proposed hypotheses, and all variables were included without constraints (Figure 3). It was found that the model fit measures were outside the acceptable levels (CMIN=10380.95, *df*=186, CMIN/*df*=55.812, CFI=0.779, SRMR=0.097, and RMSEA=0.111). We therefore started procedures to improve the model fit. Because we did not want to delete items, as proposed by an examination of the standardized residual covariances in order to preserve the established scales, we applied constraint of error variances. We ended the procedure when acceptable fits were achieved (CMIN=4068.80, *df*=179, CMIN/*df*=22.731, CFI=0.916, SRMR=0.076, and RMSEA=0.070).

The values of the regression coefficients followed the same pattern as the correlational and SEM analysis of sums, but all the values were somewhat higher. We could explain 25% of the stress using the proposed model.

**Table 4:** Correlations^a^ (Spearman *ρ*) between totals for PSS-4^b^, SAT-5^c^, and FETSOS^d^ (N=4555).

	FETSOS	SAT-5	PSS-4
FETSOS	—^e^	—	—
SAT-5	–.390	—	—
PSS-4	.316	–.361	—

^a^All correlations were significant at the 0.01 level (2-tailed).

^b^PSS-4: Perceived Stress Scale.

^c^SAT-5: Satisfaction with Online Study Scale.

^d^FETSOS: Feelings Towards Study Obligations Scale.

^e^Not applicable.

## Discussion

### Principal Findings

The long-lasting COVID-19 pandemic, with its numerous accompanying preventive measures, imposed a variety of challenges affecting the mental health of the population, with young and emerging adults, which coincides with the beginning of postsecondary education, being more significantly affected [49]. This group already faces a number of stress-related and mental health difficulties due to the characteristics of this transitional period of life, such as instability owing to changes in education, living arrangements, and relationships. COVID-19-related challenges might additionally impact the latter [49,50]. The aim of this study was therefore to examine the levels of stress of Slovenian postsecondary students during the forced online distance learning occasioned by COVID-19 lockdowns and to identify the factors influencing it.

In response to the pandemic, many countries replaced face-to-face education with distance education. This could potentially result in negative social, psychological, and academic consequences for postsecondary students [51]. The results of this study indicate that more than half of the students reported higher levels of stress, with students studying for bachelor’s degrees reporting higher levels than those studying for a master’s degree or a doctorate. This is in line with previous research, which showed higher levels of stress among students engaged in distance education [5,6,17-20,22,23,52-54]. Stress levels also differed significantly between gender groups, with females reporting higher levels of perceived stress than males. This could be attributed to the greater vulnerability of women to the development of mental health problems in general [55,56], as well during the COVID-19 pandemic [18,20,49,51,57].

Although the students who participated in our study reported the online study experience as being comprehensible, successful, and instructive, the overall satisfaction with online study was the lowest among bachelor’s degree students, with no significant differences between the gender groups. Overall, the results show 3 main factors to be related to students’ negative emotions associated with the timeliness, performance, and quality of the study obligations: a decline in the motivation to study, the quality of internet and mobile connections, and the presence of distractors in the study space (eg, other people). Once again, bachelor’s degree students reported higher levels of negative emotions associated with the aforementioned factors compared to other student groups. These findings are in line with previous reports, which show COVID-19 to have had a negative impact on the academic experiences of postsecondary students [49,58,59], with distance education resulting in higher levels of stress and isolation; a negative mood; and lower levels of relatedness, concentration, focus, motivation, and performance compared to face-to-face education [58].

All 3 initial hypotheses were tested and supported. We were able to explain 25% of stress measured by PSS-4 with the SEM full model and 17% by use of the sums of the responses. The results from correlational and regression analyses showed that the level of satisfaction with online study influences stress (H1) in such a way that the higher the satisfaction, the lower the stress (Spearman *ρ*=–.361, path coefficient=–.25). These results are in line with Lee and Jang’s findings [60], indicating a negative correlation between students’ overall stress and satisfaction scores with their study life, although their research was not specifically focused on online study.

The second hypothesis, that feelings toward online study (working conditions, pedagogy, and well-being) influence satisfaction, was also confirmed. The results (Spearman *ρ*=–.390, path coefficient=.32) suggest that more positive feelings connected with the timeliness, performance, and quality of the study obligations result in more positive satisfaction with online study and thus indirectly in lower levels of stress. These results were expected as they have been produced by previous, as well pre-COVID-19 studies [51,52].

The third hypothesis, that feelings toward online study (working conditions, pedagogy, and well-being) influence stress, was also confirmed. The results (Spearman *ρ*=–.316, path coefficient=.32) suggest that higher scores or less negative feelings connected with the timeliness, performance, and quality of the study obligations reduce stress. Similar results were found in a qualitative study conducted among university students during the COVID-19 lockdown, which showed that the quality of internet connections and the study environment were among the main sources of students’ stress [61].

The results clearly show that more positive satisfaction with online study and more positive feelings toward study obligations during COVID-19 lockdowns are moderate predictors of stress. FETSOS is a negative predictor of SAT-5, meaning that positive feelings (opinions) toward the study obligations result in higher satisfaction and that lower satisfaction and more intense negative feelings toward the study obligations are predictors of higher stress. Academic struggles may therefore increase already elevated distress among the postsecondary population [62]. Based on the findings of this study, the levels of perceived stress are higher in female and bachelor’s degree students. However, differences in the terms of effect sizes are small. The differences in FETSOS and SAT-5 in both categories are almost nonexistent in terms of effect size.

The results of this study show higher levels of perceived stress among Slovenian postsecondary students during the COVID-19 pandemic, although the fact that the survey was conducted during the exam period might also have influenced the stress levels. Moreover, an upward trend in feelings of stress was also reported before the pandemic. Data from the international research study “Mladina 2018–2019” [63] show a high increase in the share of Slovenian young people aged 14-29 years who reported, over a 5-year interval, feeling stressed most of the days of the week. Similarly, data from a national study titled “Health-Related Behavioural Style of Slovenian Residents” [64] show that approximately a quarter (23.2%) of Slovenian people aged between 25 and 74 years reported regular or daily feelings of stress, with stress more commonly reported by women and younger individuals (28.3% of those aged between 25 and 34 years). To obtain a better insight into the trend in perceived stress in postpandemic times, a longitudinal study that also includes other potential impact factors would be desirable.

Prolonged, recurrent stress and poor stress management are among the key factors in the deterioration of an individual's health, as they increase the risk of many diseases and disorders. Conversely, reducing stressors and strengthening an individual's resilience to stress make an important contribution to maintaining and improving mental health. From the findings of this study, we can therefore recommend that implementing structures and measures targeted at stress reduction, such as the establishment of psychological support, is crucial, especially in cases where stress levels call for mental health treatment. However, any measures should target working conditions and pedagogy as well, which calls for the elimination of or at least a reduction in the obstacles identified by FETSOS. It is outside the scope of this paper to suggest practical measures to postsecondary educational institutions, but those related to pedagogy are completely within its domain. At the top of the scale is motivation. This should therefore be raised (or at least not decreased) by all means possible at institutional and individual levels, showing students that the future is not dark and without hope. There is also no excuse not to make expectations about outcomes clear, and to increase access to necessary resources, at least in digital format. Organizing an e-library, for example, should not present a big problem. Problems with internet connections and adequacy of the workspace are factors that were induced by the lockdown and confinement to the home environment. Stress would also be reduced by keeping campuses and dormitories open, and by providing an online structure and ensuring that libraries are close by. However, it can be reasonably expected that due to the higher virulence (even with lower severity) of the new strains of coronavirus [65], or even the possible emergence of new zoonotic viruses [66], the experience gained over the past two years should be incorporated into efforts to minimize the negative impacts of internet-based education.

### Limitations

The main limitation of the study was the self-selection of the respondents. Even if a high number of respondents were included in the data set, they are representative of a population who responded to all of the items. It is therefore impossible to make inferences about the population of individuals who did not respond to the study. Furthermore, the fact that the study featured a preponderance of male respondents could have influenced the results, as certain mental health problems may be more frequent among female students. Although other mental health problems (anxiety, depression, etc) and other variables that interact with or influence stress are important, they were not included in any recent study and we did not report them in this paper. The missing links remain to be explored.

### Directions for Future Research

We suggest that the survey be repeated after the lockdowns have ended to find out whether there are differences in levels of stress and the factors impacting it. Additionally, an international comparative study would shed light on the differences that are based on various sociocultural factors.

### Conclusion

In conclusion, the side effects caused by FODE [3], as well as the ad hoc educational practices resulting from the pandemic [3], resulted in higher stress, which is known as 1 of the main causes of mental health problems, such as anxiety and depression. We can only hope that these effects are just transitional. If they are not, society will see a higher number of people with mental health problems well into the future.

## Appendix

Multimedia Appendix 1 Questionnaire.

Multimedia Appendix 2 Measures of central tendencies (N=4455), communalities and factor loadings of the PSS-4 (N=2235). PSS-4: Perceived Stress Scale.

Multimedia Appendix 3 Measures of central tendencies (N=4455), communalities, and factor loadings of SAT-5 (N=2235). SAT-5: Satisfaction with Online Study Scale.

Multimedia Appendix 4 Measures of central tendencies (N=4455), communalities, and factor loadings of FETSOS (N=2235). FETSOS: Feelings Towards Study Obligations Scale.

## Abbreviations

CFA: confirmatory factor analysis
EFA: exploratory factor analysis
FETSOS: Feelings Towards Study Obligations Scale
FODE: forced online distance education
PAF: principal axis factoring
PSS-4: Perceived Stress Scale
SAT-5: Satisfaction with Online Study Scale
SEM: structural equation modeling
