# Supplementary material for: Influence of Forced Online Distance Education During the COVID-19 Pandemic on the Perceived Stress of Postsecondary Students: Cross-sectional Study
Source: J Med Internet Res. 2022 Mar 15;24(3):e30778. doi: 10.2196/30778 (PMC9132369; doi:10.2196/30778)
Supplement: Multimedia Appendix 1 [file jmir_v24i3e30778_app1.docx]

**Multimedia Appendix 1: Questionnaire**

With the outbreak of COVID-19 epidemic, we have been faced with specific challenges and numerous changes to our daily routines. The epidemic has significantly impacted our lives. The resulting changes that have emerged might pose a great risk to mental and physical health, and influence our perception and behaviour. The National Institute of Public Health, in cooperation with other organisations and institutions, are carrying out and developing various activities that aim to address the changed circumstances brought about by the COVID-19 epidemic and its potential aftermath. In order to deliver the best response to the present situation and needs, we ask you to fill out the following questionnaire. We are interested in exploring how you perceived the COVID-19 epidemic, the associated measurements, stress, burdens, new formats of study and your health. By participating, you will help us to obtain an insight into the present situation, which will help us to develop effective solutions.

The present research is part of the ongoing project ‘Measurements in the field of COVID-19 spread management with specific focus on vulnerable populations’ (the investment is co-financed by Slovenia and the European Union from European social funds).

Participation in the research is entirely voluntary. We kindly ask you to answer all of the questions. Only then will we be able to obtain a full insight into the present situation. You can stop answering the questions at any point. Even if you decide that you do not wish to participate, you will not face any repercussions. All of the information obtained from this research will be entirely confidential, anonymous and kept in accordance with the Slovenian Personal Data Protection Act and the General Data Protection Regulation (GDPR). The research data is being gathered entirely for scientific research purposes, with the intention of developing support services. If you have any inquiries relating to the research, you can contact us at tajnistvo-covid19@nijz.si.

All rights reserved. No part of this document may be reproduced or forwarded in any form or in any manner, electronic or physical form, including photocopying or any data saving system or data gathering system without written permission.

Thank you for your participation.

On behalf of the team,

assist. prof. Branko Gabrovec, PhD, project manager

**Q1 – Before you begin answering the questions, please confirm your participation in the study by confirming the statements below.**


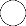
 I agree to voluntarily participate in the research and to the storage of my anonymous responses for scientific purposes. I have been apprised of the purposes of the research.

**A0 – Please indicate your student status**


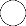
 Full-time student


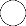
 Part-time student


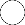
 Other:

**A1 – Indicate your sex**


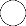
 Female


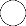
 Male


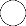
 I don’t want to answer

**A3 – What is the level of the study programme you are enrolled in?**


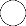
 Higher education programme


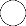
 Undergraduate or Bachelor’s degree programme


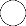
 Postgraduate Master’s programme


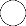
 Postgraduate doctoral programme


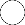
 Other:

**A5 – Please indicate your field of study**


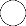
 Humanities (e.g. history and archaeology, philosophy, psychology, linguistics, literature)


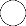
 Arts (e.g. music, film, fine arts, architecture and design)


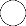
 Social sciences without education (e.g. sociology, economics, law)


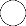
 Social sciences – education (e.g. education, didactics or subject didactics)


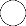
 Class teacher


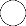
 Single-subject or double-subject teacher


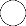
 Nursery school teacher


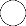
 Natural sciences and mathematics (e.g. biology, physics, chemistry, pharmacy)


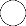
Technology and engineering (e.g. mechanical engineering, biotechnology)


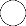
 Medicine


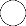
 Health sciences


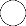
 Security sciences


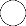
 Other:

**A8 – What is your relationship status?**


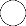
 Single


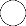
 In a partnership


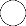
 Other:

**Q5 – Below is a set of questions related to how you are coping with the situation.**

_____________________________________________________________________

**STR1 – The questions in this scale ask you about your feelings and thoughts during THE LAST MONTH. In each case, please indicate your response HOW OFTEN you felt or thought a certain way: 1 – Never, 2 – Almost never, 3 – Sometimes, 4 – Fairly often, 5 – Very often**

|  | 1 – Never | 2 – Almost never | 3 – Sometimes | 4 – Fairly often | 5 – Very often |
| --- | --- | --- | --- | --- | --- |
| In the last month, how often have you felt that you were unable to control the important things in your life? | 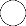 | 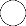 | 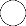 | 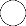 | 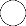 |
| In the last month, how often have you felt confident about your ability to handle your personal problems? | 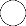 | 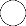 | 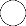 | 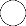 | 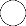 |
| In the last month, how often have you felt that things were going your way? | 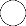 | 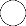 | 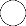 | 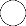 | 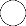 |
| In the last month, how often have you felt difficulties were piling up so high that you could not overcome them? | 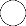 | 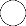 | 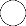 | 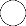 | 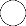 |

**Q7 – Below is a set of arguments related to distance learning.**

_____________________________________________________________________

**S1 - There are several statements listed below. Please choose an answer that most corresponds to what you believe: 1 – Completely disagree, 2 – Disagree, 3 – Partly disagree, 4 – Neither agree nor disagree, 5 – Partly agree, 6 – Agree, 7 – Completely agree**

|  | 1 – Completely disagree | 2 – Disagree | 3 – Partly disagree | 4 – Neither agree nor disagree | 5 – Partly agree | 6 - Agree | 7 – Completely agree |
| --- | --- | --- | --- | --- | --- | --- | --- |
| After undergoing distance learning, I can describe the experience as entertaining. | 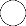 | 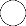 | 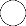 | 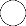 | 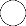 | 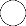 | 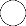 |
| After undergoing distance learning, I can describe the experience as instructive. | 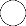 | 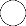 | 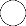 | 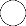 | 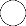 | 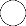 | 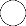 |
| After undergoing distance learning, I can describe the experience as easy. | 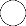 | 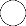 | 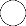 | 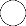 | 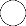 | 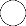 | 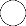 |
| After undergoing distance learning, I can describe the experience as comprehensible. | 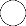 | 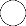 | 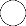 | 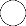 | 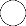 | 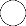 | 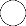 |
| After undergoing distance learning, I can describe the experience as successful. | 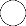 | 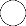 | 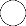 | 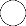 | 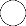 | 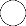 | 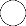 |

**S2 – There are several statements listed below. Please indicate to what extent the factors listed below influence your negative feelings relating to the performance, quality and timeliness of completion of your study obligations: 1 – No influence, 2 – Very weak influence, 3 – Weak influence, 4 – Partial influence, 5 – Strong influence, 6 – Very strong influence, 7 – Absolute influence**

|  | 1 – No influence | 2 – Very weak influence | 3 – Weak influence | 4 – Partial influence | 5 – Strong influence | 6 – Very strong influence | 7 – Absolute influence |
| --- | --- | --- | --- | --- | --- | --- | --- |
| The quality of computer and communication equipment. | 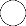 | 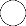 | 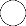 | 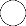 | 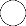 | 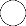 | 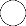 |
| The quality of internet and mobile data connection. | 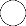 | 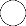 | 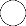 | 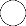 | 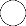 | 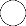 | 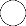 |
| Adequateness of the study space. | 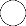 | 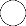 | 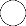 |  |  |  |  |
| Presence of attention-disruptors in the study space (e.g. presence of other people). |  |  |  |  |  |  |  |
| Household chores and other housework. |  |  |  |  |  |  |  |
| Obtaining income to make a living. |  |  |  |  |  |  |  |
| g – Access to study resources (e.g. library). |  |  |  |  |  |  |  |
| Increased study demands by teachers. |  |  |  |  |  |  |  |
| Uncertainty relating to regulations and the process of completing the study obligations that cannot be done via distance learning (e.g. internships, practical workshops/skills labs). |  |  |  |  |  |  |  |
| Health issues directly related to distance learning. |  |  |  |  |  |  |  |
| Health issues not directly related to distance learning. |  |  |  |  |  |  |  |
| Decrease in motivation to study. |  |  |  |  |  |  |  |
