# Supplementary material for: Influence of Forced Online Distance Education During the COVID-19 Pandemic on the Perceived Stress of Postsecondary Students: Cross-sectional Study
Source: J Med Internet Res. 2022 Mar 15;24(3):e30778. doi: 10.2196/30778 (PMC9132369; doi:10.2196/30778)
Supplement: Multimedia Appendix 2 [file jmir_v24i3e30778_app2.docx]

Multimedia Appendix 2: Measures of central tendencies (N = 4,455), communalities and factor loadings of the PSS-4 (N = 2,235).

|  | Mean | SD | Median | Mode | Skewness | Kurtosis | Comm | F1 |
| --- | --- | --- | --- | --- | --- | --- | --- | --- |
| STR1 | 2.35 | 1.15 | 2 | 2 | -0.22 | -0.72 | 0.61 | 0.78 |
| STR2R^a^ | 1.67 | 0.95 | 2 | 2 | 0.16 | -0.26 | 0.35 | 0.59 |
| STR3R | 1.99 | 0.93 | 2 | 2 | 0.01 | -0.32 | 0.49 | 0.70 |
| STR4 | 1.96 | 1.15 | 2 | 2 | 0.10 | -0.76 | 0.56 | 0.75 |
| STR sum | 7.97 | 3.32 | 8 | 8 | -0.04 | -0.41 |  |  |

Note. ^a^R indicates reverse scored items.
