# Supplementary material for: Influence of Forced Online Distance Education During the COVID-19 Pandemic on the Perceived Stress of Postsecondary Students: Cross-sectional Study
Source: J Med Internet Res. 2022 Mar 15;24(3):e30778. doi: 10.2196/30778 (PMC9132369; doi:10.2196/30778)
Supplement: Multimedia Appendix 3 [file jmir_v24i3e30778_app3.docx]

Multimedia Appendix 3: Measures of central tendencies (N = 4,455), communalities and factor loadings of the SAT-5 (N = 2,235).

|  | Mean | SD | Median | Mode | Skewness | Kurtosis | Comm^a^ | F1 |
| --- | --- | --- | --- | --- | --- | --- | --- | --- |
| SAT1 (entertaining) | 3.06 | 1.97 | 2 | 1 | 0.54 | -1.03 | 0.63 | 0.79 |
| SAT2 (instructive) | 3.93 | 1.91 | 4 | 5 | -0.15 | -1.19 | 0.65 | 0.80 |
| SAT3 (easy) | 2.69 | 1.78 | 2 | 1 | 0.88 | -0.36 | 0.34 | 0.59 |
| SAT4 (comprehensible) | 4.23 | 1.85 | 5 | 5 | -0.29 | -1.02 | 0.66 | 0.81 |
| SAT5 (successful) | 4.04 | 1.94 | 4 | 5 | -0.17 | -1.17 | 0.74 | 0.86 |
| SAT sum | 17.96 | 7.809 | 18.00 | 5 | 0.196 | -0.75 |  |  |

Note. ^a^Communalities.
