# Supplementary material for: Influence of Forced Online Distance Education During the COVID-19 Pandemic on the Perceived Stress of Postsecondary Students: Cross-sectional Study
Source: J Med Internet Res. 2022 Mar 15;24(3):e30778. doi: 10.2196/30778 (PMC9132369; doi:10.2196/30778)
Supplement: Multimedia Appendix 4 [file jmir_v24i3e30778_app4.docx]

Multimedia Appendix 4: Measures of central tendencies (N = 4,455), communalities and factor loadings of the (N = 2,235) of FETSOS.

| ID | Theoretical construct | Item | Mean | Med | Mod | SD | Skewness | Kurtosis | Communalities | F1 | F2 |
| --- | --- | --- | --- | --- | --- | --- | --- | --- | --- | --- | --- |
| S2l | Pedagogy | Decline in motivation to study | 5.37 | 6 | 7 | 1.86 | -1.02 | -0.05 | 0.36 | 0.52 |  |
| S2b | Working conditions | Quality of internet and mobile connections | 5.32 | 6 | 7 | 1.76 | -0.93 | -0.09 | 0.73 | 0.70 | -0.49 |
| S2d | Working conditions | Presence of distractions in the study space (e.g. other people) | 4.97 | 5 | 7 | 1.81 | -0.69 | -0.52 | 0.55 | 0.72 |  |
| S2c | Working conditions | Adequacy of the workspace | 4.91 | 5 | 6 | 1.79 | -0.73 | -0.37 | 0.62 | 0.75 |  |
| S2i | Pedagogy | Uncertainty with rules and procedures for completing obligations that could not be performed remotely (exercises, practice) | 4.75 | 5 | 7 | 1.95 | -0.55 | -0.83 | 0.38 | 0.57 |  |
| S2a | Working conditions | Quality of computer and communication equipment | 4.72 | 5 | 5 | 1.84 | -0.57 | -0.62 | 0.73 | 0.71 | -0.48 |
| S2g | Pedagogy | Access to study resources (e.g. library) | 4.62 | 5 | 5 | 1.8 | -0.41 | -0.78 | 0.39 | 0.60 |  |
| S2h | Pedagogy | Increased study demands from teachers | 4.53 | 5 | 4 | 1.76 | -0.35 | -0.69 | 0.41 | 0.61 |  |
| S2e | Working conditions | Household and other chores | 4.25 | 4 | 4 | 1.76 | -0.17 | -0.83 | 0.44 | 0.66 |  |
| S2f | Well-being | Need to earn an income | 3.88 | 4 | 1 | 2.12 | 0.05 | -1.31 | 0.34 | 0.57 |  |
| S2j | Well-being | Health problems directly related to distance learning | 3.30 | 3 | 1 | 2.00 | 0.38 | -1.08 | 0.53 | 0.61 |  |
| S2k | Well-being | Health problems not directly related to distance learning | 2.95 | 3 | 1 | 1.86 | 0.62 | -0.68 | 0.38 | 0.52 |  |
| total |  |  | 53.56 | 55 | 60 | 14.68 | -0.36 | -0.17 |  |  |  |
